# 5-Methyltetrahydrofolate and Vitamin B12 Supplementation Is Associated with Clinical Pregnancy and Live Birth in Women Undergoing Assisted Reproductive Technology

**DOI:** 10.3390/ijerph182312280

**Published:** 2021-11-23

**Authors:** Michela Cirillo, Rossella Fucci, Sara Rubini, Maria Elisabetta Coccia, Cinzia Fatini

**Affiliations:** 1 Department of Experimental and Clinical Medicine, University of Florence, 50134 Florence, Italy; michela.cirillo@unifi.it; 2 Center for Assisted Reproductive Technology, Division of Obstetrics and Gynecology, Careggi University Hospital, 50134 Florence, Italy; fuccir@aou-careggi.toscana.it (R.F.); sara.rubini@studio.unibo.it (S.R.); mariaelisabetta.coccia@unifi.it (M.E.C.); 3 Department of Clinical and Experimental Biomedical Sciences, University of Florence, 50134 Florence, Italy

**Keywords:** assisted reproduction, folate, vitamin B complex, dietary supplement, MTHFR, pregnancy

## Abstract

The homocysteine pathway in the preconception period should be evaluated to highlight micronutrient deficiencies and warrants optimal multivitamin supplementation, before Assisted Reproduction, as preconception care. We conducted a retrospective study aimed at investigating the role of vitamin B complex (5-methyltetrahydrofolate, vitamin B12, vitamin B6) supplement use compared with the role of only folic acid supplement use, in relation to clinical pregnancy and live birth in infertile women undergoing homologous ART. We investigated 269 Caucasian women referred to the Centre for Assisted Reproductive Technology for homologous ART. In these women, 111 (Group A) were daily supplemented with vitamin B complex and 158 (Group B) with only folic acid. In group A the mean number of Metaphase II oocytes and the 2PN Fertilization Rate were higher in comparison to group A (*p* = 0.04; *p* = 0.05, respectively). A higher percentage of women in group A had a clinical pregnancy and live birth in comparison to group B (*p* = 0.01; *p* = 0.02, respectively). Vitamin B complex supplementation remained independently associated, after multivariable adjustment, with clinical pregnancy (OR 2.03, *p* = 0.008) and live birth (OR 1.83, *p* = 0.03). Women supplemented with 5-MTHF and vitamin B12, have a higher chance of clinical pregnancy and live birth in comparison to those supplemented with only folic acid.

## 1. Introduction

Assisted Reproductive Technologies (ART) represent widely used procedures for the control and treatment of infertility, despite that the majority of procedures remain unsuccessful, and the reason of this lack of success may have a multifactorial origin. Therefore, the need to identify modifiable predictors of successful infertility treatments may be of relevance. Among these predictors, folate and vitamin B12 represent an interesting investigation target which could positively influence reproductive success after ART [1,2]. Folate is metabolized through the one-carbon metabolism cycle and genetic factors impact the cycling and availability of active byproducts. Folic acid is the synthetic form incorporated into foods and prenatal supplements, and has no biological activity until it is reduced into the bioactive folate derivative, 5-methyltetrahydrofolate (5-MTHF). The gene encoding the MTHFR enzyme has a key role in converting folate to its biologically active form, 5-methyltetrahydrofolate. Common MTHFR genetic variants alter enzyme function and folate metabolism efficiency. The impact of these components in regulating homocysteine metabolism (folate, vitamin B12, vitamin B6 and vitamin B2) has been studied in the physiology of human reproduction. Nevertheless, there are no univocal data about the role of folate in improving success rates in couples undergoing ART [3,4]; to date, women receiving folic acid supplementation had better quality oocytes and a higher degree of mature oocytes compared with those without supplementation [1]. Folate plays a role in DNA synthesis and epigenetic modification, as well as cell proliferation. Therefore, folate deficiency increases the risk of neural tube defects (NTDs) and other complex congenital malformations. Furthermore, some studies have shown an association between vitamin B12 deficiency and reduced fertility [5,6] as well as embryo quality [7]; however, correlation between vitamin B12 levels and pregnancy outcome after ART are not conclusive [3,8]. Finally, data from a clinical trial suggest that supplementation with vitamin B6 in infertile women is able to increase reproductive performance, with a 40% increase in the probability of conception and a 30% reduction in early pregnancy loss [9].

The shortage of these vitamins results in hyper-homocysteinemia, which is believed to have important embryotoxic effects, and adverse obstetric/neonatal outcomes [10], beyond women’s atherothrombotic risk. In particular, elevated homocysteine levels have been associated with an increase in spontaneous pregnancy loss [8,11], and a defect in vascularization of the chorionic villi [12]. Furthermore, the levels of homocysteine in the follicular fluid seem to be inversely related to oocyte maturity and embryo developmental capabilities in vitro [13,14]. Therefore, to test for homocysteine, vitamin B status as well as MTHFR polymorphisms in the preconception period should be useful in highlighting micronutrients deficiencies and warrant an optimal multivitamin supplementation before ART as preconception care.

Based on these observations, the purpose of this study was to investigate the role of vitamin B complex (400 µg 5-methyltetrahydrofolate, 5 µg vitamin B12, 3 mg vitamin B6) supplement use compared with the role of only folic acid (400 µg) supplement use, in relation to pregnancy outcomes (clinical pregnancy, pregnancy loss and live birth) in infertile women undergoing homologous ART.

## 2. Materials and Methods

### 2.1. Study Population

In this retrospective study, we investigated Caucasian women referred to the Internal Medicine Clinic (which consists of a multidisciplinary team focused on the prevention of cardiovascular risk) at the Centre for Assisted Reproductive Technology, Careggi University Hospital (Florence, Italy) from May 2018 to April 2020, for homologous ART. Included in the study were 269 women supplemented with vitamin B complex (400 µg 5-methyltetrahydrofolate, 5 µg vitamin B12, 3 mg vitamin B6) or only folic acid (400 µg) (according to gynecologist’s prescription).

### 2.2. Covariate Assessment

Cardiovascular risk factors, as well as history of negative obstetric events were investigated during the clinical evaluation. Information concerning gynecological diseases (Polycystic Ovary Syndrome (PCOS), endometriosis), C677T and A1298C polymorphisms of the MTHFR gene, thrombophilia, Anti-Müllerian Hormone (AMH), Follicle Stimulating Hormone (FSH) and Antral Follicle Count (AFC) were derived from clinical reports.

According to the WHO criteria, overweight was defined as BMI values 25–29.99 kg/m^2^. Dyslipidemia was defined according to European Society of Cardiology (ESC) guidelines [15]. The women were considered to have hypertension if they had been diagnosed as hypertensive according to ESH/ESC guidelines (≥140/90 mm Hg) or were taking antihypertensive drugs [16]. Migraine with aura was diagnosed by physician according to The International Classification of Headache Disorders 3rd edition [17]. Physical activity grade was investigated and sedentary behavior was defined as absent or light (i.e., either occasional walking or recreational activity only). Recurrent pregnancy loss (RPL) was defined as the loss of two or more pregnancies [18] and recurrent implantation failure was defined according to Bashiri et al. [19].

Total homocysteine (free and bound to proteins), vitamin B6, Vitamin B12 and folate concentrations were derived from clinical reports before starting folic acid and vitamin B complex supplement.

Examinations for HIV 1/2, HCVab, HbsAg, HbsAb, HBcAb were performed in both partners.

Informed written consent for anonymous data analysis was obtained from all women. The study was conducted according to the guidelines of the Declaration of Helsinki, and approved by the Ethics Committee of Azienda Ospedaliera Universitaria-Careggi (Florence) (11292-04/12/2017).

### 2.3. Laboratory Assessment

#### Sperm Processing

Semen samples were obtained after 2–7 days of sexual abstinence and collected into sterile plastic containers. After liquefaction ejaculates were analyzed and classified [20]. Semen quality parameters considered were sperm concentration, motility and morphology. Semen samples were processed under sterile conditions using the density gradient method with 95%, 70% and 50% gradient layers (PureSperm^®^100, Nidacon International AB; Mölndal, Sweden) heated in the incubator set at 37 °C for 30 min. About 1 mL of liquefied sample was gently overlaid on the gradients and centrifuged at 300× *g* for 20 min. The supernatant was carefully aspirated and discarded using a sterile Pasteur pipette for each tube and the pellet was moved into a new conical tube, re-suspended in 2.0 mL of medium (Flushing, Origio, Cooper Surgical Fertility & Genomic Solutions, Malov, Denmark) and centrifuged again at 200× *g* for 10 min. After centrifugation, the supernatant was discarded using a disposable sterile Pasteur pipette and the pellet was re-suspended in 0.5 mL of fertilization medium (Origio, Cooper Surgical Fertility & Genomic Solutions, Malov, Denmark). We assessed the sperm count and motility of the washed pellet under sterile conditions. This pellet was stored for later use in the ART procedure.

### 2.4. Clinical Procedures and Outcome Assessment

Couples underwent ART with ICSI (Intracytoplasmic sperm injection). The ovarian stimulation began with 150–225 IU of recombinant FSH (Gonal-F^®^; Merck Serono; Darmstadt, Germany) from day 2 of the menstrual cycle, and the GnRH antagonist (Cetrotide; Merck Serono, Darmstadt, Germany) was introduced according to a multiple-dose protocol (0.25 mg/day), once a leading follicle of 14 mm and/or estradiol concentrations of 400 pg/mL were reached. The triggering was performed when at least three follicles > 17 mm were present with 0.2 mg of triptorelin SC (Decapeptyl, Ipsen Pharma, Paris, France), or recombinant human chorionic gonadotrophin (HCG) (Ovitrelle^®^, Merck Serono Europe Limited, London, UK) and oocyte retrieval was performed under sedation at the 36th hour following the triggering. In the same day of oocyte retrieval, all the women started the luteal phase therapy support with daily 400 mg intravaginal capsules (Progeffik^®^/Proimetrium^®^) and progesterone 25 mg IM injection (Pleyris^®^). Embryo transfer was performed at day 3.

Follicular fluid obtained from the eggs retrieval was observed under stereomicroscope (NIKON, SMZ 1500) in sterile condition in a vertical laminar hood at 37 °C. During the retrieval, the oocytes were placed in 1 mL drops of Fertilization medium (Origio, Cooper Surgical) in a 60 mm dishes under an oil (Vitrolife) overlay. At the end of the retrieval, the excess of cumulus cells was cut with a pair of insulin syringes, and the oocytes were placed, in groups of three–five, in 20 µL drops of Fertilization medium (Origio, Cooper Surgical).

Following 2–3 h of incubation at 37 °C with 6% CO2 and 5% of O_2_, hyaluronidase (Sage) was used to assist in denudation of cumulus cells surrounding the oocytes. Intracytoplasmic sperm injection (ICSI) was then used to inseminate all mature oocytes.

After microinjection, in the ICSI cycles, oocytes were cultured overnight in incubators at 37 °C with 6% CO_2_ and 5% O_2_ in preequilibrated 20 µL drops of GTL (Vitrolife) under an oil (Vitrolife) over- lay.

After 12–18 h of culture, the oocytes were assessed for fertilization.

The normal fertilized oocytes (2PN) were then transferred into fresh drops of the GTL (Vitrolife) and placed in incubators at 37 °C with 6% CO_2_ and 5% of O_2_.

Embryos with the highest cell number and the highest grade were selected for embryo transfer.

Pregnancy was confirmed by a positive result in a urinary human chorionic gonadotropin test. Gestational sac was observed ultra-sonographically 5 weeks after embryo transfer, corresponding to 7 weeks of pregnancy. Clinical pregnancy was defined as the presence of a gestational sac. Miscarriage was defined as pregnancy loss after the presence of a verified gestational sac, but before 20 weeks of gestation. Live birth was defined as delivery of a child after ART.

#### Statistical Analysis

Data are reported as frequency (%) and as mean ± SD. The non-parametric Mann–Whitney test for unpaired data was used for comparisons of continuous variables between single groups. Chi-square test was used to test for proportions. Univariate and multivariate analysis adjusted for covariates (age (<40 yrs.), BMI 25–29.99 kg/m^2^, smoking habit, dyslipidemia, antithrombotic therapy) were used in order to evaluate the association between vitamin B complex supplementation and clinical pregnancy and live birth. Odds ratios and 95% confidence intervals were presented. A *p*-value of less than 0.05 was considered to indicate statistical significance.

Based on a prevalence of clinical pregnancy of about 45% in women supplemented with folic acid who underwent homologous ART, post hoc sample size calculation indicated that at least 98 women supplemented for each group were sufficient for detection purposes, with a statistical power of 85% (β) and a significance value of 0.05 (α), and a prevalence of about 60% for clinical pregnancy. All statistical analyzes are performed using statistical software (SPSS, version 27.0 for Windows; SPSS, Chicago, IL, USA).

## 3. Results

A total of 269 infertile women participated in this study, mean age 36.9 (±3.7) yrs. We observed that 111 women (Group A) were daily supplemented with vitamin B complex (400 µg 5-MTHF, 5 µg vitamin B12, 3 mg vitamin B6) and 158 women (Group B) supplemented with only folic acid (400 µg folic acid). In terms of markers of ovarian reserve, mean AMH was 2.9 (±3.2) ng/mL, mean FSH was 8.6 (±4) IU/mL and mean AFC was 10.1 (± 6.9).

No significant differences in AMH, FSH and AFC between the two groups investigated, were found.

The evaluation of traditional CV risk factors evidenced that about 23.4% of women had a BMI 25–29.99 kg/m^2^ (24.3% group A vs. 22.8% group B), 26% of women had smoking habit (27.9% group A vs. 24.7% group B), and 66.9% of women had sedentary behavior (70.3% group A vs. 64.6% group B). Dyslipidemia was present in 47.6% of women, in particular in 44.1% of women in group A and in 50% of women in group B. Regarding obstetric history, 37.2% had at least two implantation failures after ART (35.1% group A vs. 38.6% group B) and 14.5% had a history of recurrent pregnancy loss (≥2) (10.8% group A vs. 17.1% group B) (Table 1).

In Table 2 inherited thrombophilia and MTHFR gene polymorphisms of investigated women are reported. No statistically significative differences were found between groups. As regards homocysteine and vitamins concentrations, no statistically significative differences between the two groups were found (homocysteine: Group A 10.2 µmol/L vs. Group B 8.6 µmol/L), (Folate: Group A 10.9 ng/mL vs. Group B 13.5 ng/mL; Vitamin: B6 Group A 12.9 µg/L vs. Group B 10.3 µg/L; Vitamin B12: Group A 368 pg/mL vs. Group B 397 pg/mL).

### 3.1. Embryology Laboratory Outcomes

**Figure 1:**
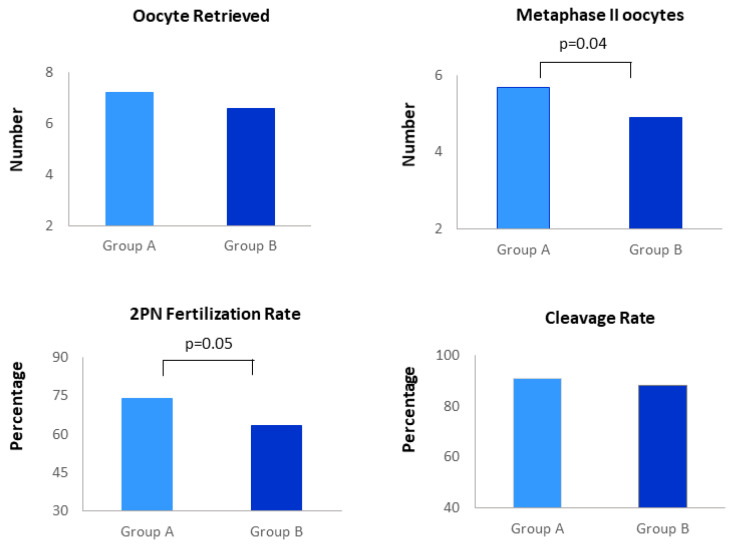
Distribution of embryological outcomes in women undergoing Assisted Reproduction supplemented with vitamin B complex (Group A) and folic acid (Group B).

When investigating early ART endpoints (Figure 1), Group A mean number of Metaphase II (MII) oocytes as well as 2PN Fertilization Rate (FR) were higher in comparison to Group B (5.7 ± 3.2 vs. 4.9 ± 4.1, *p* = 0.04; 74.1% vs. 63.5%, *p* = 0.05, respectively). No other associations emerged (Oocytes retrieved 7.2 ± 3.9 vs. 6.6 ± 4.6; Cleavage Rate 91% vs. 88%) (Figure 1). As concern semen quality parameter (assessed by quantification of sperm concentration, motility and morphology), no statistically significant differences in partners of Group A and B were found.

### 3.2. Pregnancy Outcomes

**Figure 2:**
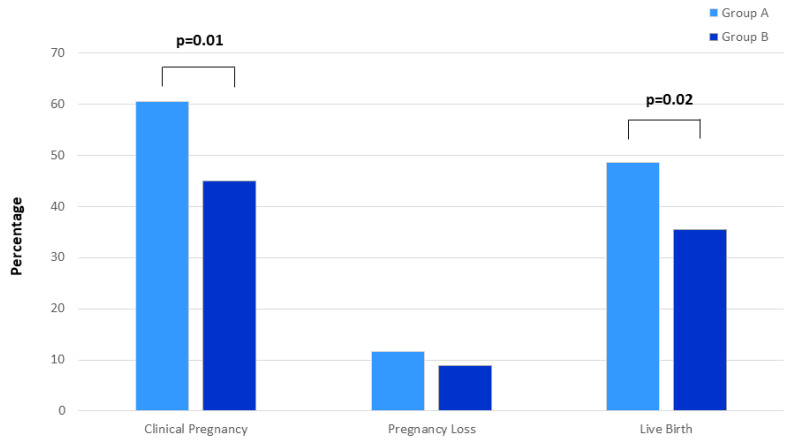
Distribution of pregnancy ART outcomes (percentage of clinical pregnancy, pregnancy loss and live birth) in women undergoing Assisted Reproduction supplemented with vitamin B complex (Group A) and folic acid (Group B).

Pregnancy outcomes are reported in Figure 2. A higher percentage of Group A women had a clinical pregnancy and live birth in comparison to Group B (60.4% vs. 44.9%, *p* = 0.01; 48.6% vs. 35.4%, *p* = 0.02, respectively). No statistically significant difference of miscarriage between the two groups was found (11.7% vs. 8.9%).

Vitamin B complex supplementation was significantly associated with clinical pregnancy and live birth (*p* = 0.01 and *p* = 0.02, respectively) (Figure 2), also after adjustment (OR=2.03, *p* = 0.008 and OR=1.83, *p* = 0.03, respectively). At multivariable analysis performed in both groups (A and B), only age < 40 years was associated with clinical pregnancy but not with live birth (Table 3).

**Figure 3:**
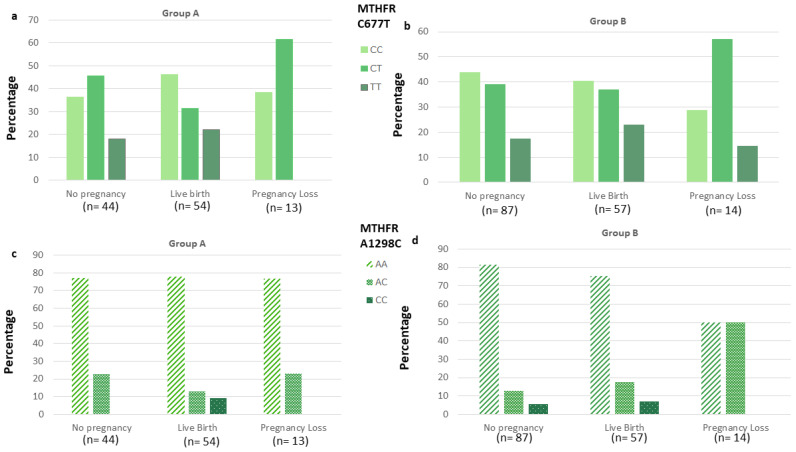
Distribution of MTHFR C677T and A1298C genotypes according to pregnancy ART outcomes (no pregnancy, live birth and pregnancy loss) in women supplemented with vitamin B complex (Group A; **a**,**c**) and with folic acid (Group B; **b**,**d**). *p* > 0.05 for distribution of MTHFR C677T and A1298C genotypes according to ART outcomes in group A and B.

We further analyzed the distribution of two polymorphisms in MTHFR gene (C677T and A1298C) in relation to clinical outcomes (clinical pregnancy, live birth and pregnancy loss) in the two groups (Figure 3). No statistically significant differences related to clinical outcomes were observed (*p* > 0.05 for all MTHFR genotypes). In Table 4, we reported data concerning homocysteine concentration at baseline for each MTHFR gene polymorphism, and ART outcomes in each group (A and B). No significant differences were observed.

## 4. Discussion

Our findings evidence a significant association between vitamin B complex supplementation and pregnancy outcomes (clinical pregnancy and live birth). Moreover, women supplemented with vitamin B complex had a higher number of MII oocytes as well as 2PN FR, than women supplemented with folic acid, thus hypothesizing a higher chance of achieving pregnancy.

Reproductive success is highly influenced by the female pre-conceptional health, including nutrition and micronutrient levels, which may play a pivotal role in this initial phase. To date, the effect of folic acid in preventing NTDs is very convincing and international guidelines [21] recommended it as a preconception supplement (400 µg/die), whereas the beneficial role of folate and Vitamin B12 on pregnancy outcome in ART women, as well as its role in modulating fertilization and embryo quality, remains undefined.

Data from clinical studies, suggest that high concentrations of some micronutrients, such as vitamin B12 and folate before ART, were associated with a better fertilization, implantation and evolution of pregnancy, thus representing an easily modifiable therapeutic target in women undergoing ART [3,7,22]. Conversely, according to other studies, there is no positive correlation between pregnancy outcomes after ART and circulating folate levels [8,23].

Based on evidence from clinical study and the knowledge that ART may increase atherothrombotic risk in women [24], we investigated the markers of vascular health in women, such as homocysteine pathway, thrombophilia, lifestyle habits and traditions, and cardiovascular risk factors specific to women. Folate and vitamin B12 are implicated in homocysteine homeostasis as well as unhealthy lifestyle, smoking, medication use, renal and liver function, endocrine and physiological processes and polymorphisms in MTHFR gene [25]. We observed that 15% of women had hyper-homocysteinemia and a high percentage were heterozygotes (C677T 36.4%; A1298C 17.5%) and homozygotes (C677T 18.6%; A1298C 5.2%) for rare alleles of MTHFR polymorphisms, beyond high percentage of smokers (26%). In women carried these variants, the supplementation with 5-MTHF, vitamin B12 and vitamin B6, might be associated to reduction in homocysteine levels and increased pregnancy outcomes [26].

Hyper-homocysteinemia, through cytotoxic and oxidative stress, may lead to impaired oocyte maturation and embryo development [27,28]. Moreover, exposure of trophoblast cells to elevated homocysteine may also increase cellular apoptosis and lead to inhibition of trophoblastic function, which is essential for successful placentation [3,29]. Folate and homocysteine are thought to be present in the follicular fluid [30], proportionally to the circulating levels. If folate and homocysteine are present in the microenvironment of the maturing oocyte, it is possible that an excess of homocysteine, or a deficiency of folate, could compromise oocyte competence in development and early embryogenesis.

Our findings showed that women supplemented with vitamin B complex (Group A) had an increase in both oocytes retrieved at pick-up and MII oocytes than that observed in Group B (only folic acid supplementation). This allowed the embryologists to obtain a higher number of oocytes for performance of ART. As a consequence, an increased number of MII oocytes is related to higher probability of obtaining 2PN oocytes, and thus possible improvement of embryo development.

Data from Heindrickx et al. [31], reported that ICSI results in an average FR of 70% and, in particular, data from recommendations of Expert Panel [32] reference values for ICSI normal FR were competence ≥ 65% and benchmark ≥ 80%. Our findings evidenced that vitamin B complex supplement intake increases oocyte competence and improves FR in Group A, overcoming minimum reference value of oocyte competence. In addition, vitamin B complex supplement intake increases Cleavage Rate, defined as the proportion of zygotes which cleaved to become embryos, in Group A vs. Group B, with a value of Cleavage Rate in keeping with alpha Survey competence value, ranging from 80–95% [32].

This study found that vitamin B complex supplement intake has a positive impact on pregnancy outcome (clinical pregnancy and live birth) following ART, independently of variables such as smoking habit, inherited trombo-philia, and BMI, known to influence ART outcomes. The role of vitamin B complex has been previously shown in studies involving ART women [3,7,22] and it must be recognized that available data are very heterogeneous for the investigated women, methodology and aim. Data from Boxmeer et al. [7] performed in women undergoing homologous ART evidenced that high folate, but not vitamin B12 concentrations in mono-follicular fluid, were correlated with embryo quality and increased chances of achieving biochemical pregnancy. The role of folate and vitamin B12 in increasing the chance of live birth after ART has been indicated by Gaskins et al. [3]; the study also suggests that serum folate and vitamin B12 may exert their favorable effects on pregnancy maintenance after implantation. High serum and red blood cells folate, but not vitamin B12, were associated with pregnancy rate. This datum was evidenced by Paffoni et al., in women undergoing IVF [22]. Conversely, high folate status before pregnancy increases the chance of twin birth, but not the likelihood of a successful pregnancy after IVF [8].

Finally, data from Murto et al. showed that women with unexplained infertility undergoing ART had better folate status than fertile women, but did not show that this would have a positive effect on pregnancy outcomes [23]. However, in this study information concerning dietary supplementation derived from self-reporting, thus representing a bias. Another study from the same group [4] investigated the association between MTHFR gene variants and pregnancy outcomes. Our data are in keeping with those from Murto, demonstrating no effect of these genetic variants on pregnancy outcomes (live birth and pregnancy loss). On the other hand, the role of most commonly studied MTHFR variants (677C > T and 1298A > C) in successful pregnancy outcome still remained intriguing.

Our study is novel as there are no other studies evaluating the relationship between vitamin B complex supplementation and embryology and pregnancy outcomes, compared with folic acid supplementation in women undergoing ART.

The limitations of this study are worth noting. First, we did not evaluate baseline dietary characteristics which would influence serum folate and vitamin B12 concentrations. On the other hand, baseline serum vitamin concentrations were similar between the two groups. Second, our data is not generalizable to other countries which use fortified food supply and high use of supplements [3]. Third, the retrospective nature of the research itself does not permit definitive and conclusive inferences, but supports the plausibility of supplementation with vitamin B complex in improving clinical pregnancy and live birth.

Another limitation is the lack of information on mono-follicular fluid vitamin concentrations, but this datum appears irrelevant, as previously reported [30].

Despite these limitations, our study has a potential strength, suggesting the possibility to evaluate early endpoints that cannot be observed in couples attempting to conceive naturally.

## 5. Conclusions

The present study shows that women undergoing homologous ART supplemented with 5-MTHF and vitamin B12, have a higher chance of clinical pregnancy and live birth in comparison to those supplemented with only folic acid. Further prospective studies and clinical randomized trial are needed to elucidate the effects of folate, vitamin B12 and homocysteine pathway in improving pregnancy outcomes in women after ART. If our findings were confirmed, this relatively inexpensive supplementation with vitamin B complex might be considered in clinical practice, in particular in women undergoing ART.

## Figures and Tables

**Table 1:** Clinical characteristics of study population.

Variables	Group A, n = 111	Group B, n = 158
Age yrs	37.4 (±3.3)	36.6 (±4)
BMI 25–29.99 kg/m^2^, n, (%)	27 (24.3)	36 (22.8)
Smoking habit, n (%)	31 (27.9)	39 (24.7)
Dyslipidemia, n (%)	49 (44.1)	79 (50)
Hypertension, n (%)	1 (0.9)	4 (2.5)
Sedentary behaviour, n (%)	78 (70.3)	102 (64.6)
Migraine with aura, n (%)	2 (1.8)	10 (6.3)
History of recurrent pregnancy loss ≥ 2, n (%)	12 (10.8)	27 (17.1)
History of recurrent ART failure ≥ 2, n (%)	39 (35.1)	61 (38.6)
Endometriosis, n (%)	14 (12.6)	23 (14.6)
PCOS, n (%)	6 (5.4)	15 (9.5)
Celiac disease, n (%)	-	-
Hashimoto’s thyroiditis, n (%)	6 (5.4)	5 (3.2)
Family history of CV disease, n (%)	32 (28.8)	46 (29.1)

Group A: vitamin B complex supplementation; Group B: Folic acid supplementation. Values are reported as mean ± SD and n (%). ART (Assisted Reproductive Technology); BMI (Body Mass Index); MetS (Metabolic Syndrome); PCOS (Polycystic Ovary Syndrome); CV (cardiovascular).

**Table 2:** Inherited thrombophilia and MTHFR gene polymorphisms.

Variables	Group A, n = 111	Group B, n = 158
Homocysteine > 13 µmol/L Factor V Leiden heterozygotes, n (%)	17 (15.3)	23 (14.6)
	9 (8.1)	13 (8.2)
Prothrombin G20210A mutation heterozygotes, n (%) PC, PS, AT deficiency, n (%)	4 (3.6)	8 (5.1)
	4 (3.6)	4 (2.5)
MTHFR C677T, CC, n (%) CT, n (%) TT, n (%)	46 (41.5) 45 (40.5) 20 (18)	65 (41.1) 63 (39.9) 30 (19)
MTHFR A1298C AA, n (%) AC, n (%) CC, n (%)	87 (78.4) 19 (17.1) 5 (4.5)	121 (76.6) 28 (17.7) 9 (5.7)

Group A: vitamin B complex supplementation; Group B: Folic acid supplementation. Values are reported as mean ± SD, n (%). ART (Assisted Reproductive Technology); PC (Protein C); PS (Protein S); AT (Antithrombin); MTHFR (Methylenetetrahydrofolate reductase).

**Table 3:** Multivariate analysis for determinants of clinical pregnancy and live birth in Group A and Group B.

Variables	Group A		Group B	
	Clinical Pregnancy			
	OR (95% CI)	*p*	OR (95% CI)	*p*
Age < 40 yrs.	3.12 (1.09–9.85)	0.05	4.91 (1.47–16.45)	0.01
Smoking habit	0.40 (0.15–1.08)	0.07	0.99 (0.39–2.49)	0.9
Dyslipidemia	0.97 (0.35–2.68)	0.9	1.48 (0.69–3.19)	0.3
BMI 25–29.99 kg/m^2^	1.36 (0.36–5.09)	0.6	1.11 (0.48–2.56)	0.8
Inherited thrombophilia	0.82 (0.16–4.35)	0.8	1.92 (0.51–7.19)	0.3
MTHFR polymorphisms	0.57 (0.17–1.91)	0.4	1.81 (0.79–4.17)	0.2
Antithrombotic therapy	1.34 (1.09–4.56)	0.6	1.42 (0.61–3.28)	0.4
	**Live Birth**			
	**OR (95% CI)**	***p***	**OR (95% CI)**	***p***
Age < 40 yrs	1.37 (0.47–4.02)	0.6	3.04 (0.92–10.08)	0.07
Smoking habit	0.64 (0.24–1.68)	0.4	1.05 (0.42–2.67)	0.9
Dyslipidemia	0.81 (0.31–2.09)	0.7	1.13 (0.52–2.44)	0.8
BMI 25–29.99 kg/m^2^	2.43 (0.70–8.38)	0.2	1.34 (0.58–3.12)	0.5
Inherited thrombophilia	0.99 (0.19–5.06)	0.9	1.73 (0.48–6.22)	0.4
MTHFR polymorphisms	1.52 (0.50–4.55)	0.5	1.09 (0.48–2.49)	0.8
Antithrombotic therapy	1.29 (0.39–4.19)	0.7	1.95 (0.81–4.67)	0.1

**Table 4:** Homocysteine concentrations for each MTHFR polymorphisms according to pregnancy ART outcomes (Live Birth, Pregnancy Loss and No Pregnancy).

	Group A (n = 111)			Group B (n = 158)		
	Live Birth (n = 54)	Pregnancy Loss (n = 13)	No Pregnancy (n = 44)	Live Birth (n = 57)	Pregnancy Loss (n = 14)	No Pregnancy (n = 87)
	Homocysteine Concentrations					
**MTHFR C677T**				**MTHFR C677T**		
CC	11.6 (±6.2)	9.4 (±2.7)	12.5 (±8.8)	9 (±3.4)	14.8 (±13.3)	8.2(±3.2)
CT	8.8 (±4.2)	8 (±1.4)	8.2 (±2.3)	8.4 (±3.5)	7.6 (±2.0)	8.1 (±2.3)
TT	12.7 (±5.9)	-	8.4 (±5)	8.3 (±4.2)	8.6 (±4.2)	9.3 (±2.5)
**MTHFR A1298C**				**MTHFR A1298C**		
AA	11.6 (±6.3)	7.9 (±1.4)	10 (±6.7)	8.6 (±3.6)	13.1 (±8.5)	8.4 (±2.9)
AC	8.1 (±0.9)	12.2 (±0.3)	9 (±3.7)	9.3 (±4.4)	6.3 (±1.2)	8.7 (±2.1)
CC	9.7 (±1.0)	-	-	7.7 (±1.8)	-	7.9 (±3.5)

Group A: vitamin B complex supplementation; Group B: Folic acid supplementation. Values are reported as mean ± SD. *p* > 0.05 for homocysteine concentrations according to pregnancy outcomes for each MTHFR gene polymorphism in group A and B.

## Data Availability

Not applicable.
